# Targeting gut microbiota to regulate the adaptive immune response in atherosclerosis

**DOI:** 10.3389/fcvm.2025.1502124

**Published:** 2025-01-31

**Authors:** Despina Giakomidi, Ayoola Ishola, Meritxell Nus

**Affiliations:** ^1^ Cardiovascular Division, Department of Medicine, Heart and Lung Research Institute (HLRI), University of Cambridge, Cambridge, United Kingdom; ^2^ British Heart Foundation Centre of Research Excellence, University of Cambridge, Cambridge, United Kingdom

**Keywords:** atherosclerosis, gut microbiota, T cells, B cells, metabolites

## Abstract

Atherosclerosis, the leading cause of death worldwide, is a chronic inflammatory disease leading to the accumulation of lipid-rich plaques in the intima of large and medium-sized arteries. Accumulating evidence indicates the important regulatory role of the adaptive immune system in atherosclerosis during all stages of the disease. The gut microbiome has also become a key regulator of atherosclerosis and immunomodulation. Whilst existing research extensively explores the impact of the microbiome on the innate immune system, only a handful of studies have explored the regulatory capacity of the microbiome on the adaptive immune system to modulate atherogenesis. Building on these concepts and the pitfalls on the gut microbiota and adaptive immune response interaction, this review explores potential strategies to therapeutically target the microbiome, including the use of prebiotics and vaccinations, which could influence the adaptive immune response and consequently plaque composition and development.

## 1 Introduction

Cardiovascular diseases (CVDs) are the leading cause of death globally (1). In the majority of cases the underlying cause is atherosclerosis, a complex arterial pathology with multiple genetic and environmental risk factors. Atherogenesis is initiated in response to the trapping of low-density lipoproteins (LDL) in the intima and their acquisition of immunogenic properties through both enzymatic and oxidative modifications. The subsequent immune response involves interactions between many vascular and circulating cells and mediators, and frequently leads to a chronic inflammatory state due, at least in part, to defects in counter-regulatory mechanisms. Extensive evidence supports the inflammatory theory of atherosclerosis (2), and innate and adaptive immune cells have been shown to participate in all stages of the disease from its initiation to progression and plaque rupture or erosion (3). Moreover, atherosclerosis is a metabolic inflammatory disease, sensitive to changes in diet and strongly influenced by the intestinal microbiota.

Microbiota, refers to the collective microbial community inside and on the surface of the human body and plays a very important role in immune homeostasis and atherosclerosis. Several studies have determined the innate immune system as a link between gut dysbiosis and atherosclerosis, but less is known about the contribution of adaptive immunity to the process. In this review we are going to focus on how the gut microbiota influences the adaptive immune response and atherosclerosis, and how it could be modulated and targeted to alter the adaptive immune response to treat atherosclerosis.

### 1.1 Atherosclerosis and adaptive immune system

The adaptive immune response is a specialized response activated through molecules (antigens) recognized by specialized, highly selected and clonally developed receptors, like immunoglobins (Ig) in B cells and T cell receptors (TCR) in T cells. Innate immune cells, mainly macrophages and dendritic cells (DC), act as antigen presenting cells (APC) and initiate the adaptive immune response. They drive the polarization of naïve CD4^+^ and CD8^+^ T cells to effector and/or memory cells of specialized T helper (Th) or T regulatory (Tregs) -cell subsets through exposure of antigenic peptides on major histocompatibility complex (MHC) class I or II molecules, along with the engagement of co-stimulatory pathways and the action of different cytokines in secondary lymphoid organs (4). While Tregs have been proposed to have an atheroprotective role, Th cells exhibit different roles depending of the subtype (5). Th1 are atherogenic cells, while Th2, Th17 and Tfh may play contextual dependent atherogenic or atheroprotective roles. All types of CD4^+^ T cells have been found in atherosclerotic plaque lesions or the adventitia as well as in the blood of patients with atherosclerotic lesions (6).

Similarly, B cells are also important players in the adaptive immune response in atherosclerosis. There are different B cell subsets with different pro-atherogenic or atheroprotective functions depending on the main antibody type or the cytokine they secrete [reviewed in detail in (7)]. In general B1 (8, 9) and B2-Marginal Zone B cells (MZB) (10–12) cells protect from atherosclerosis by secreting IgM antibodies in a T independent or dependent manner respectively. While B2-Follicular B (FOB) cells are considered atherogenic for promoting the formation of the Germinal Centre (GC) (13) response and atherogenic class switched IgG antibodies.

### 1.2 Gut microbiota

The human body contains a broad number of microorganisms (∼4 × 10^13^ microbial cells) including bacteria, viruses, protozoa, archaea and fungi, which constitute the commensal microbiota that mainly resides in the gut. This commensal flora is unique to each individual and has a mutualistic relationship with its host. On one hand, it benefits from a constant supply of host substrates and in return, the host also benefits from bacterial activities that are important to keep a homeostatic physiological body balance (14) like fermentation of non-digestible substrates (i.e., dietary fibres); production of certain vitamins (i.e., vitamin E); maintenance of the correct functioning of the immune system; protection against infections; as well as, controlling the gut-brain communication network between the enteric and the central nervous system. Gut microbiota express over 3 × 10^6^ of genes producing millions of metabolites with diverse functions in the host. Collectively, these genes expressed by the microbiota constitute the microbiome (15, 16). There are several factors that can modulate gut microbiota like host genetics, age and diet. Based on the Twins UK study (17) less than 10% of the gut microbiota *taxa* may exhibit a heritable trait, thus in general environmental factors like diet are more important determinants of the microbiota composition. Several mouse and human studies have reproducibly shown that a more diverse gut microbiota is associated with a “healthy gut”, conversely, a dramatic imbalance in the composition and function of these microorganisms, termed as gut dysbiosis, leads to a decreased microbial diversity that translates into a “leaky gut” and systemic inflammation causing obesity, autoimmune diseases, type 2 diabetes and cardiovascular diseases (18). Many studies have demonstrated that a diverse gut can be achieved with a diverse diet (19), but other environmental factors like medication and blood clinical markers also have an important role. For example, in a recent large-scale human study integrating microbiota profiles with clinical blood markers, diet and medication, blood LDL levels were strongly negatively associated with gut diversity (20).

There are five major bacterial phyla in the intestinal flora: *Bacteroidetes (*includes genera like *Bacteroides* and *Prevotella)*; *Firmicutes* [includes genera like Clostridium (∼95%), *Lactobacillus*, *Bacillus*, *Enterococcus,* and *Ruminicoccus]*; *Actinobacteria* (includes genera like *Bifidobacterium)*; *Proteobacteria*; *Fusobacteria* and *Verrucomicrobia* (21) that have distinct functions and are predominantly located in specific regions along the gut. In the lower intestine anaerobic bacteria are the predominant type particularly *Bacteroides*, *Bifidobacteria*, *Fusobacteria* and *Peptostreptococci,* while anaerobes and facultative aerobes such as *Enterobacteria* and *Lactobacilli* are present at moderate density (22). In homeostasis, more than 90% of bacteria in both mice and human consist of *Bacteroidetes* and *Firmicutes* (23). An increased *Firmicutes*/*Bacteroidetes* (*F*/*B*) ratio has been associated with obesity (24) and CVDs (25), so for many years, this ratio has been widely considered to be a marker of gut health. However, this concept has been challenged by more recent studies on obesity [reviewed in (26, 27)] and the same may apply to CVD, which has been lesser studied than obesity but further experiments are necessary to corroborate this.

### 1.3 Gut microbiota and atherosclerosis

Gut microbiota does regulate the risk factors (dyslipaemia, hypertension, obesity etc) [extensively reviewed in (28–30)] and the regulators (immune and inflammatory cells and mediators) [extensively reviewed in (31)] that lead to atherosclerosis. Thus, there is no surprise that studies in germ-free animals (GF) have shown that the gut microbiota plays a prominent role in atherosclerosis. Unexpectedly, GF *ApoE^−/−^* mice, with higher plasma and hepatic cholesterol levels, developed less atherosclerosis than conventionally raised (Conv) *ApoE^−/−^* fed a control diet (early atherosclerosis model) (32, 33). Conversely, there were no significant differences when feeding a high fat high cholesterol (HF HC) diet for 12 weeks to the same mice models (33) or for 16 weeks to Conv and GF *LDLr^−/−^* (advanced atherosclerotic models) (34). These experiments suggest that gut microbiota may play a more significant role in early atherosclerosis, a time-point in which the adaptive immune response plays a more prominent role (35). Thus, further experiments are necessary to explore how gut microbiota could be modulating the adaptive immune response and its effect on early atherosclerosis.

In humans there are a very few and small-sized studies that have been performed to identify gut microbiota species that are different between individuals with atherosclerosis or other CVD and healthy controls (Table 1) (36–55). Bacterial DNA resembling that in the gut is present in atherosclerotic plaques, but apart from a handful of descriptive studies there are not functional studies to understand the role of these bacteria in the plaque and if they could be associated with increased CV risk (41, 53). Many different gut species have also been identified as significantly increased or decreased in patients vs. controls, but there is no consensus regarding species that are directly linked to increased risk (36–55). Furthermore, while decreased *alpha*-diversity is associated with disease in general, only one study comparing controls and atherosclerotic patients found increased diversity in healthy vs. atherosclerotic patients (), so clearly larger epidemiological studies are needed to shed light into the relationship between gut microbiota and CVD. Using a considerable sample from the Framingham Heart Study it was shown that microbial diversity decreased with 10-year CVD risk, and this was mostly driven by BMI and lifestyle factors (54).

**Table 1:** List of clinical studies determining atherosclerotic plaque or gut microbiota composition in individuals with atherosclerosis and other associated cardiovascular diseases vs. healthy controls. CE, carotid endarectomy; SCA, subclinical carotid atherosclerosis; CAD, coronary artery disease; CAS, carotid atherosclerosis; A, atherosclerosis; CHD, coronary heart disease; CVD, cardiovascular disease; IHD, ischemic heart disease; AP, atherosclerotic plaques; F, faecal; 16S, 16S rRNA sequencing; MS, metagenomic shotgun sequencing; P, patients; C, control; TMAO, trimethylamine-N-Oxide; LPS, lipopolysaccharide; HDL-C, high density lipoprotein—cholesterol; FHS, framingham heart study. TC, total cholesterol; LDL-C, low density lipoprotein; SCFAs, short-chain fatty acids; NS, not significant differences; NM, not measured. Terminal restriction fragment length polymorphism (T-RFLP).

Condition	Country	Sample	Anal	*N*	Gut microbiota in patients vs. controls	Clinical parameters	*α* diversity
CE (36)	Denmark	AP	MS	15SP vs. 7C	• ↓*Porphyromonadaceae, Bacteroidaceae, Micrococcaceae, Streptococcaceae* • ↑*Helicobacteraceae* (*H. pylori*), *Neisseriaceae* (*N. polysaccharea*), *Thiotrichaceae, Acinetobacter spp, Acidovorax spp*		NM
CE (37)	Sweden	AP	16S	12SP vs. 15AP	• ↑*Allobaculum, Erycipelotrichaceae, Erysipelotrichales, B. elkanii* • ↓*Coeynebacteriaceae, Corynebacterium*		NM
SCA (38)	Italy	F	16S MS	144P vs. 201C	• ↑*Enterobacteriaceae (Escherichia, Shigella), Firmicutes [Oscillospira, Streptococcus (S. salivarius, S. parasanguinis, S. anginosus), Ruminococcus (R. obeum), Lactobacillaceae (L. gasseri, L. fermentum), Dorea (D. longicatena), Clostridium (C. leptum), Eubacteriaceae (Eu. ramulus], Lachnospiraceae (Coprococcus), Parabacteroides (Pa. goldsteinii*) • ↓*Bacteroides (B. uniformis, B. thetaiotaomicron), Ruminococcus (R. bromii), Firmicutes (F. prausnitzii)*	• ↑TMAO, LPS • ↓Butyrate	NM
CAD (39)	China	F	16S	70P vs. 98C	• ↓Firmicutes (Faecalibacterium, Roseburia, Eubacterium, Clostridium, Lachnospiraceae*, Ruminococcaceae), Oscillospiraceae (Subdoligranulum, Flavonifractor) • ↑Firmicutes (Phascolarctobacterium**), Enterobacteriaceae (Escherichia, Shigella) Lactobacillaceae (Lactobacillus), Enterococcaceae (Enterococcus), Streptococcaceae (Lactococcus), Erycipelotrichia (Catenibacterium), Bacillus, Leuconostocaceae), Pseudomonadaceae (Pseudomonas)	• ↓Butyrate, HDL-C • ↑TMAO*, TC**, LDL-C**, HDL-C	NM
SCA (40)	Hungary	F	16S	14 twins	• ↑*Firmicutes [Lachnospiraceae (Roseburia), Ruminococcacceae (Faecalibacterium)), Bacteroidaceae (Bacteroides), Actinobacteria, Eubacteriales (Blautia)* • ↓*Bacteroidetes, Prevotellaceae*	•	NS
CAS (41)	Sweden	F	MS	12P vs. 13C	• ↑*Firmicutes (Ruminococcus), Actinobacteria (Collinsella)* • ↓*Firmicutes (Eubacterium, Roseburia)*	•	NM
A (42)	China	F	MS	218P vs. 187C	• ↑Enterobacteriaceae [E. coli, K. pneumoniae, K. oxytoca, E. aerogenes], Firmicutes (Streptococcus spp, L. salivarius, S. moorei, R. gnavus, unclassified Erysipelotrichaceae*, C. nexile*, S. anginosus*), Actinobacteria (A. parvulum, E. lenta, B. dentium) • ↓Firmicutes (R. intestinalis, F. prausnitzii), Bacteroides (P. copri, A. shahii)	• ↑LPS, TMAO* • ↓Lipid A synthesis	NS
CAD (43)	Japan	F	(T-RFLP)	39P vs. 30C vs. 50H	• ↑Firmicutes (Lactobacillales, Clostridium) • ↓Bacteroidetes (Bacteroides, Prevotella)	•	NM
SCA (44)	China	F	MS	569P	• ↑Firmicutes (Enterococcus, Turicibacter), Euryarcheota (Methanobrevibacter), Proteobacteria (helicobacter), Actinobacteria (Libanicoccus) • ↓Firmicutes (Faecalicatena), Bacteroides (Alistipes), Proteobacteria (Acinetobacter, Oligella)	• ↓Butyrate	Shannon index measured in multiple factors
SCA (45)	China	F	16S	32P vs. 32C	• ↑Firmicutes (Acidaminococcus, Christensenella, Lactobacillus) • ↓Firmicutes (Anaerostipes, Fusobacterium, Gemella, Parvimonas, Romboutsia, Clostridium)	•	NS
CHD (46)	China	F	16S	29P vs. 35H	• ↑Firmicutes (Clostridia), Bacteroides, Fusobacteria • ↓Proteobacteria, Bacteroidetes (Bacteroidia)	•	NS
CAD (47)	China	F	16S	161P vs. 40C	• ↓Firmicutes [Lachnospiraceae Roseburia), Ruminococcaceae (Faecalibacterium)] • ↑Firmicutes (Veillonella), Proteobacteria (Haemophilus, Klebsiella)	• ↓Butyric acid • ↑LPS	NS for SCAD and controls ** for UA and MI
CAS (48)	China	F	MS	31P vs. 51C	• ↓Bacteroidetes (Prevotellaceae), Proteobacteria (Pasteurellaceae, Haemophilus, E. coli**, Halomonas unclassified**, K. pneumoniae, Pantoea unclassified), Firmicutes (A. defectiva, A. intestini, G. haemolysans, L. mucosae, L. lactis, M. elsdenii, R. sp JC304, S. anginosus, T. sanguinis, Turibacter unclassified) • ↑Bacteroidetes (B. sp 3_1_19, P. unclassified, P copri*)	• ↓SCFAs, LPS*, TMAO** • ↑LPS, SCFAs	NS
CVD (49)	India	B	16S	80P vs. 4° C	• ↑Actinobacteria (Propionibacteriaceae, Corynebacterium, Rhodococcus, Mycobacterium, Bifidobacterium, Brachybacterium, Clavibacter, Nocardia, Kocuria, Mobiluncus, Arthrobacter, Actinobacillus, Acinetobacter, Streptomyces, Cellulomonas, Leifsonia), Firmicutes (Streptococcus, Bacillus, Acidaminococcus, Micrococcus), Bacteroidetes, Chlorobi, Chrloroflexi, Cyanobacteria, Acidobacteria, Deinococcus-Thermus, Gemmatimonadetes, Thermotogae, Rhizobiales, Rhodobacterales, Enterobacteriales (Sinorhizobium, Myxobacterium, Escherichia, Bradyrhizobium, Methylobacterium) • ↓Proteobacteria (Pseudomonadaceae, Pseudomonas, Rhodopseudomonas, Escherichia, Shigella, Paracoccus)	•	NM
CVD (50)	USA	F	16S	55HR vs. 50LR	• ↑Bacteroidetes (Prevotella, Bacteroidales), Firmicutes (Tyzzerella, Coprococcus) Proteobacteria (Enterobacter, Thalassospira), Euryarcheota (Methanobrevibacter) • ↓Bacteroidetes (Paraprevotella, Alloprevotella), Firmicutes (Megamonas, Catenibacterium, Megasphaera, Ruminococcus, Christensenellaceae)	•	NS
Twins (51)	UK	F	16S	617 women	• ↓Ruminococcaceae, Rikenellaceae, Clostridiaceae, Collinsella aerofaciens, Barnesiellaceae, Odoribacter	•	NS
IHD (52)	Denmark France Germany	F	MS	372P vs. 275C	• ↓Proteobacteria (Acinetobacter, T. muris, Acetobacter, P. excrementihominis), Bacteroidetes (B. intestinalis, C. secundus, Alistipes unclassified), Firmicutes (Clostridiales unclassified, R. callidus, Dorea, C. comes, L. rogosae, R. timonensis, Lachnoclostridium unclassified, Butyricicoccus unclassified, E. ramulus), Actinobacteria (Eggerthella) • ↑Betaproteobacteria (B. pseudomallei), Firmicutes (M. timinensis, Lachnoclostridium unclassified, oscillibacter unclassified, E. tayi, S. intestinalis, M. faecis, C. leptum, C. symbiosum, H. hathewayi 2), Proteobacteria (Burkholderiales unclassified), Bacteoidetes (B. clarus), Actinobacteria (C. bouchesdurhonensis, B. dentium)		NM
Stroke (53)	China	F	16S	141P vs. 94C	• ↑Proteobacteria [Enterobacter, Deltaproteobacteria (Desulfovibrionales, Desulfovibrionaceae, Desulfovibrio)], Bacteroidetes (Porphyromonadaceae, Parabacteroides, Rikenellaceae, Alistipes), Firmicutes (Megasphaera, Lactobacillus, Lactobacillaceae, Eubacteriaceae, Eubacterium, Oscillibacter, Subdoligranulum), Synergistaceae • ↓Proteobacteria (Shewanella, Shewanellaceae), Bacteroidetes (Bacteroidales, Bacteroidia, Paraprevotella, Prevotella, Prevotellaceae), Firmicutes (Faecalibacterium, Anaerosporobacter)	• ↓TMAO	NS
FHS (54)	USA	F	16S	1423	• ↑Ruminococcus, Sutterella, Roseburia, Clostridiales, Dorea • ↓Lachnospiraceae, Oscillospira, Blautia producta, Bilophila	•	NS
FHS (55)	USA	F	MS LC-MS	1429	• ↑Oscilibacter (Alistipes), Ruminococcus (C. bolteae, F. plautii, B obeum, B vulgatus, C. clostridioforme), Parabacteroides merdae, Firmicutes, Methanobrevibacter smithii, • ↓Oscilobacter (Alistipes obesi)	• ↑dicarboxylic acids • ↑γBB (TMAO)	

**Figure 1:**
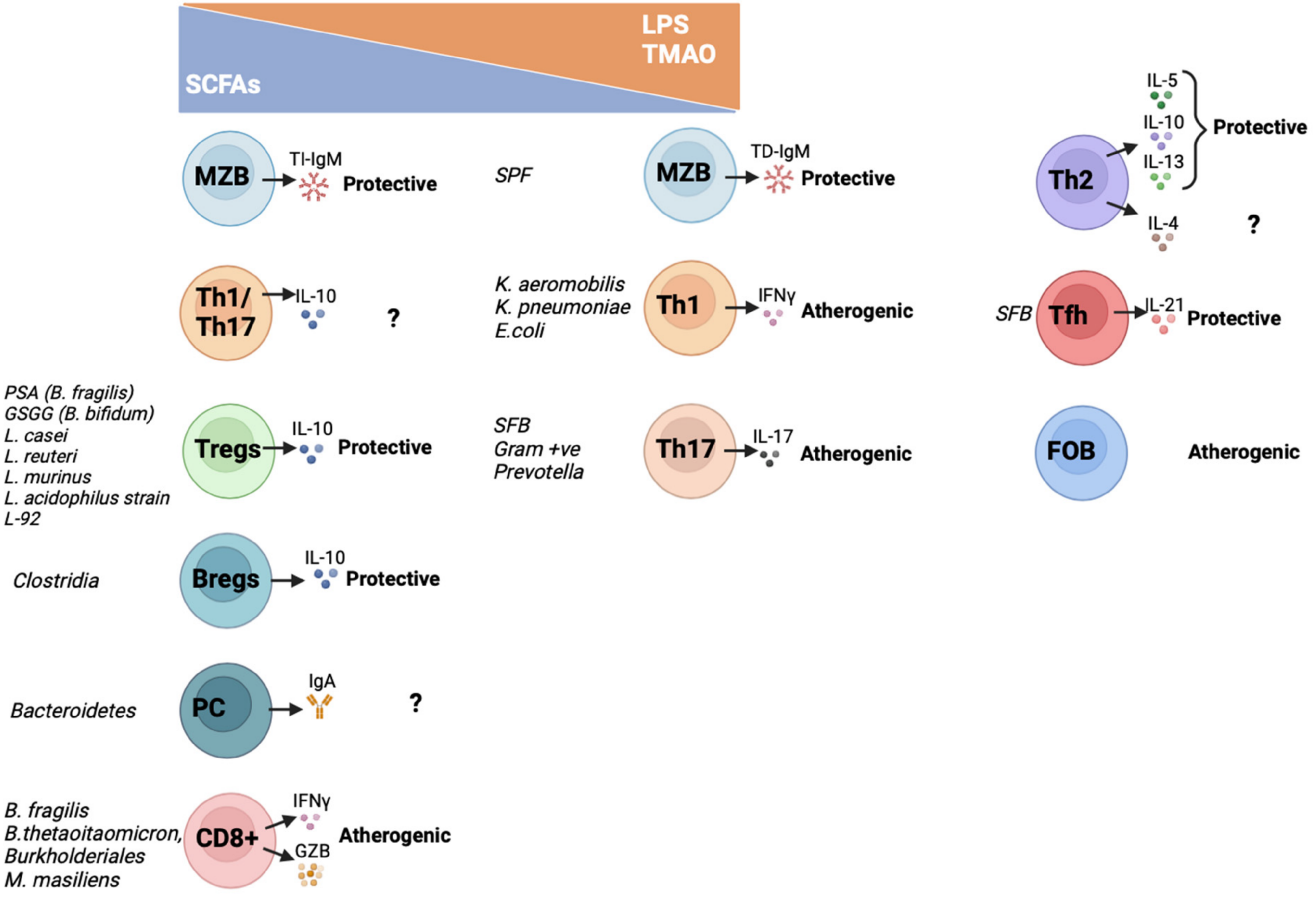
Interactions between metabolites, gut microbiota and adaptive immune cells and their effect on atherosclerosis. In this graph it is summarized how gut microbiota species and derived metabolites regulate activation and cytokine and antibody secretion of the different adaptive immune cell subsets. On one hand, atheroprotective SCFA increase TI-IgM secretion by MZB cells; IL10-Th1/Th17 secreting cells; Tregs; Bregs; IgA-producing PC and CD8T cells. On the other hand, pro-atherogenic TMAO and/or LPS activate TD-IgM secretion by MZB cells; Th1 and Th17 cells. The metabolites that activate Th2, Tfh and FOB have not been characterized yet. SCFAs, short chain fatty acids; LPS, lipopolysaccharide; TMAO, trimethylamine N-oxide; MZB, Marginal Zone B cells; Th, T helper cells; Tfh, T follicular helper cells; Tregs, T regulatory cells; Bregs, B regulatory cells; PC, plasma cells; FOB, Follicular B cells; PSA, polysaccharide A; GSGG, beta-glycan/galactan; SFB, Segmented filamentous bacteria; SPF, specific pathogen free; TI-IgM, T-independent immunoglobulin M; TD-IgM, T-dependent immunoglobulin M; IgA, immunoglobulin A; IL, interleukin; IFNγ, interferon γ; GZB, granzyme B.

Despite not specific species have been identified, there is growing evidence on how specific gut microbiota products and metabolites are linked to increased risk of atherosclerosis (p.e. LPS and TMAO), while others exhibit atheroprotective properties [p.e. short chain fatty acids (SCFA)] [reviewed extensively in (57, 58)]. These metabolites also influence the adaptive immune response, an in general those atherogenic metabolites favor proinflammatory subsets while atheroprotective metabolites enhance anti-inflammatory adaptive immune cells (Figure 1) as it will be summarised below.

### 1.4 Gut microbiota and the adaptive immune response and its potential role on atherosclerosis

For many years, special attention was paid to the role of the gut microbiota in the gastrointestinal tract and associated tertiary lymphoid structures. More recently, it has been shown that the gut microbiota exerts a remote effect, and may affect the immune response systemically (59, 60). One reason for this is that gut microbiota releases active metabolites and molecules (p.e. LPS, TMAO or SCFA) that can interact with remotely located organs and affect systemic immune responses and atherosclerosis. Another reason for this is because immune cells that change locally due to diet and gut composition in mesenteric lymphoid nodes migrate to the periphery fueling T cell accumulation within atherosclerotic lesions (61). Bellow we will summarize how the different immune cell subsets can be modulated by gut microbiota locally and/or systemically and how this can impact the development of atherosclerosis.

#### 1.4.1 Cd4+ T cells

##### 1.4.1.1 Th1 cells

Th1 cells express the transcription factor *T-bet* and signal transducer and activator of transcription 4 (STAT-4), and secrete interferon γ (IFN- γ), interleukin 2 (IL-2), IL-3, tumour necrosis factor (TNF) and lymphotoxin (62, 63). Experimental mouse models have shown that Th1 cells are pro-atherogenic [reviewed in (5)] and are the dominant CD4^+^ T cells in human atherosclerotic lesions (64–66).

Several gut bacteria species (*p.e. Klebsiella* and *E. coli* strains from the *Enterobacteriaceae* family) have been shown to locally modulate Th1 polarization in both mice (67) and humans (68). In accordance with this, *Enterobacteriaceae* were also significantly increased in the *faeces* of different atherosclerotic cohorts compared to healthy controls (38, 39, 42, 49, 50) that could potentially contributted to the development of the disease. As expected, atheroprotective SCFAs like butyrate were shown to inhibit *T-bet* and IFNγ (42), skewing Th1 differentiation into anti-inflammatory IL-10-secreting in a G-coupled protein receptor (GPR)-43 dependent-manner in experimental mouse models of colitis and in human T cells (69). On the contrary, pro-atherogenic LPS and TMAO were shown to enhance the polarization of pro-inflammatory macrophages resulting in the expansion and proliferation of Th1 and Th17 cells in advance mouse models of atherosclerosis (70, 71).

##### 1.4.1.2 Th2 cells

Th2 cells express the transcription factor *GATA3* and secrete IL-4, IL-5, IL-10 and IL-13 (72). Because Th2 signature cytokines can counteract the Th1 pro-inflammatory response, they were initially considered protective in atherosclerosis. But now we know, that while IL-5, IL-10 and IL-13 are atheroprotective, controversial results have been found regarding IL-4 [reviewed in (5, 73)]. As expected, those species that enhance a Th1 response have been shown to limit a Th2 response (p.e. *Lactobacillus* strains and *B. fragilis)* (67, 74).

##### 1.4.1.3 Th17 cells

Th17 cells expressing the transcription factor RAR-related orphan receptor (*ROR*) γt, are activated by IL-23 and secrete IL-17. Their role in atherosclerosis remains controversial as both atherogenic (75, 76) and atheroprotective (77) effects have been described.

Th17, especially those located in the intestine, are among the T cells that are more amenable by the gut microbiota and their interactions have been widely studied in autoimmune and metabolic diseases. In fact, mouse studies have shown that the gut microbiota is essential for Th17 differentiation (78, 79). Colonization of GF mice intestine with segmented filamentous bacteria (SFB), gram-positive bacteria and *Prevotella* induced Th17 differentiation and promoted secretion of IL-17 and IL-22 (67, 80–83). In a similar manner to Th1, SCFA inhibit RORγt and Th17 differentiation (74) and promote Th17 IL-10-secreting cells through inhibition of histone deacetylase (HDAC) and activation of mTOR in a GPR-43 independent manner (84). In atherosclerosis, *LDLr^−/−^* mice fed a HF/HC diet supplemented with a cocktail of peptides that can modify the growth from a HF/HC diet derived gut microbiota toward a low-fat diet one, lead to a significant increase of Tregs and a decrease of Th17 reducing atherosclerosis (85).

##### 1.4.1.4 Tfh cells

Tfh cells express the transcription factor *Bcl-6* and the chemokine receptors CXCR5, ICOS and PD1. They are a specialized subset of CD4^+^ T cells that provide help to B cells and are essential for germinal center formation, affinity maturation and the development of high affinity antibodies and memory B cells (86). They are atheroprotective by modulating IgM secretion of MZB cells (10–12).

The relationship between gut microbiota and Tfh is mutual, not only does gut microbiota affect Tfh differentiation and function but Tfh also shapes gut microbiota through receptors that are able to sense the gut microbiota and to produce an appropriate ecosystem for its development. So, on one hand *P2X7* (87) and PD1 (88) on Tfh are necessary to secrete gut microbiota specific IgA antibodies and to maintain a more diverse microbiota. And on the other hand, Tfh differentiation are absent in GF mice and restored upon microbial transplant in a TLR2-MyD88 dependent manner (89). Also, SFB in Peyer's patches were shown to enhance pro-inflammatory Tfh differentiation by restricting IL-2 access to CD4^+^ T cells in a dendritic cell dependent manner and favoring *Bcl-6* expression in the gut of a mouse model of arthritis (90).

##### 1.4.1.5 Treg cells

Treg cells express the transcription factor *FoxP3* and secrete anti-inflammatory cytokines like IL-10, TGF-*β*, and IL-35 (91). They preserve immune tolerance, block excessive inflammation and have an immune suppressive activity (92). They exhibit a prominent atheroprotective role (93–96) and clinical trials using low dose IL-2 (which increases Tregs) have been initiated to treat ischaemic heart disease (97, 98). Interestingly, low dose IL-2 has been shown to affect gut microbiota in mice and humans (99) so this interaction may have important therapeutical implications.

Both intestinal and peripheral Treg cells differentiation are regulated by SCFA in a GPR-43-dependent manner (100). Moreover, butyrate and propionate enhance extrathymic Treg production activating intronic enhancer *CNS1* (101) or inhibiting HDAC (101, 102) respectively. As expected, in atherosclerosis, *ApoE^−/−^* mice fed a HF/HC diet supplemented with propionic acid developed less atherosclerotic plaques than those that were not supplemented due to increased Treg cell numbers and lL-10 levels in the gut microenvironment (103). And these effects were reverted when blocking IL-10R. The promising beneficial effects of propionic acid are all based in animal studies, thus an exhaustive toxicity and safety study should be run before being able to think in translational studies. Other microbiota derived metabolites like polysaccharide A produced by *B. fragilis* (104) and beta-glycan/galactan produced by *B. bifidum* also promote expansion of Treg cells and IL-10 production in a TLR2 signalling dependent manner (105). Several specific strains, like *Lactobacillus casei* (106), *L. reuteri* (107), *L. murinus* (108) and *L. acidophilus* strain *L-92* (109) have been associated with increased production of Tregs in small experimental animal models.

#### 1.4.2 Cd8^+^ T cells

Similarly to CD4^+^ T cells, CD8^+^ T cells are generated in the thymus and express the TCR but they recognize antigens presented by MHC class I (located in all nucleated cells). They participate in the host defense against intracellular pathogens and tumor surveillance. They secrete effector cytokines like IFNγ and TNF*α* but they also have a cytotoxic function by secreting perforins and granzymes (GZ). In human plaques it has been reported that their percentage is lower than CD4^+^ T cells but, as the disease advances, and specifically in lesions susceptible to rupture, they account for up to half of the T cell population (110–112). Experimental animal models and single cell transcriptomics of human atherosclerotic plaques have shown that GZB and GZK producing CD8^+^ T cells enhance the development of atherosclerosis (112–114).

CD8^+^ T cells are reduced in GF mice, and several species have been shown to enhance their activation (102, 115–117). Controversial results have been shown about the impact of butyrate and propionate on CD8^+^ T cells, some showing an inhibitory effect (118), while others an stimulatory effect by enhancing their IFNγ and GZB secretion in a GPR-41/43 independent manner inhibiting HDAC (115). Thus, experimental studies supplementing with SCFA to increase Tregs will need to check that they do not enhance proatherosclerotic CD8+ T cells.

#### 1.4.3 B lymphocytes

B cells play important roles in both innate and adaptive immune responses. They undergo hypersomatic mutations and become antibody-producing cells (GC, plasmablasts and plasma cells). Antibodies are glycoproteins of the Ig family that are attached to the B cell membranes serving as B cell receptor (BCR) for antigens or can be secreted into the extracellular space and the circulation where they bind to auto- or foreign antigens. Several studies have shown that bacterial antigens are essential for the maturation, differentiation and antibody secretion of all B cells.

##### 1.4.3.1 Follicular B cells (FOB)

FOB cells are B2 cells that are located in the follicles in the spleen and can circulate in both mice and humans between other secondary lymphoid organs like Peyer's patches in the gut (119). They produce class switch antibodies in a T-cell dependent manner through the activation of BCR, TLR and BAFF/APRIL signaling pathways. They are activated in the follicles by Tfh entering the GC response and generating PC and high affinity antibodies (IgG, IgA, IgE etc). Both, FOB and GC B are considered proatherogenic (120–123).

sIgA are the first line of defense in protecting the mucosal tissues from infections and maintaining gut homeostasis within the microbiota (124–126). They are produced by B cells in the Peyer patches and are induced by food antigens and gut bacteria [p.e. gut microbiota derived SCFA and *Bacteroidetes* are indispensable to generate IgA producing PC by inhibiting HDAC and boosting PC differentiation signalling pathways (like Xbp1) (127)]. Very little is known about the potential role of IgA in atherosclerosis. On one hand, ApoE*^−/−^* atherosclerotic mice have higher IgA levels than *C57Bl6* WT mice (128). And in humans, high levels of sIgA in blood have recently been associated with increased severity of atherosclerosis in the Rotterdam Study (129). But on another hand, sIgA also have atheroprotective properties like maintaining a diverse microbiota and facilitating Tregs activation (130). Thus, mouse and human studies are needed to clarify the exact role of sIgA in atherosclerosis.

Gut microbiota also has an important role in regulating the rest of the Ig repertoire besides IgA, but it has been less studied. Using *ApoE^−/−^* mice treated with high spectrum antibiotics, Chen et al. demonstrated that gut microbiota is an important trigger for the recruitment and activation of pro-atherogenic B2 cells producing IgG in perivascular fat (131). Further studies are needed to establish the importance of gut microbiota in antibody class switching.

##### 1.4.3.2 Marginal zone B cells (MZB)

MZB cells reside only in the outer layer of the follicles in the spleen in mice. But in humans unswitched MZ-like cells account for the majority of the activated B cells in blood (132). We have previously shown that in response to a high fat high cholesterol (HF/HC) diet, MZB cells protect from atherosclerosis by limiting Tfh cells in a Pdl1-dependent manner downstream of TLR and BCR signalling pathways (10). LPS levels were significantly increased in *LDLr^−/−^* fed a HF/HC so we hypothesize that gut microbiota is necessary to activate MZB cells atheroprotective programme and further experiments are needed to test if the absence of TLRs specifically in MZB cells would affect their activation and function in the atherosclerotic context.

MZB cells are not affected in GF mice, but several studies have shown that gut microbiota is important in their development and antibody secretion. Mice with a restricted flora (RF) (rich in *Firmicutes*) show a complete deletion of MZB cells while their development was normal in SPF mice (rich in *Bacteroidetes*) (133). Regarding their BCR repertoire, human MZB cell precursors migrate to the gut associated lymphoid tissue (GALT) for somatic hypermutation and this process is regulated by gut commensals (134, 135). Furthermore, T independent IgM secretion by MZB cells depend on the presence of peri-MZ neutrophils that colonize that area after post-natal microbial colonization (136) as well as on GPR43, as its deletion in MZB cells, decreases expression of surface proteins (CD21, CD1d, CD24, IgM, etc) and enhances the formation of T-independent IgM antibodies and anti-dsDNA autoantibodies (137).

##### 1.4.3.3 B1 cells

In mice B1 cells reside in the peritoneum and the pleural cavities and are divided into B1-a and B1-b cells. Their human counterparts have started to be identified (138). Experimental mice models have shown that B1 cells protect from atherosclerosis in a T cell independent manner by generating natural IgM antibodies (8, 9, 139). Although B1 cells can also migrate to the intestines, their contribution to the total IgA plasma pool is minimal as demonstrated using gnotobiotic Ig allotype chimeric mice (140). Similarly, to MZB cells, commensal microbe and post-natal microbial colonization drives the pre-immune B cell repertoire of B1 cells and the concomitant development of IgA and IgM secreting PC (141, 142). It has been described that in aging there is an accumulation of pro-inflammatory B1 cells that express 4-1BBL leading to insulin resistance due to a significant decrease of anti-inflammatory *A. muciniphila* in the gut (143).

##### 1.4.3.4 B regulatory (Breg) cells

Breg cells exert immunosuppressive and regulatory functions. They increase in response to proinflammatory IL-6, IL-1β, IL-21, BAFF and GM-CSF and secrete IL-10 (144). In general, they exhibit an atheroprotective role (145), despite it might be through other functions independently of IL10 secretion (146).

Gut microbiota is necessary for splenic and MLN Breg cells differentiation as well as IL-10 and IL-35 secretion (147). Mice with aberrant or lack of gut microbiota do not develop IL-10-secreting Breg cells in an IL-6/IL-1B (147) and a TLR2/TLR9 ligands dependent manner (148, 149). As expected, *Clostridia* (that is the main strain that produces SCFA) induces the secretion of IL-10 by Bregs (150).

### 1.5 How we could target the gut microbiota to treat atherosclerosis regulating the adaptive immune response

#### 1.5.1 Probiotics

Probiotics are live microorganisms that are beneficial for the host's health when administered in sufficient amounts (151). They can be used to combat gut microbiome dysbiosis, inhibiting the growth of harmful bacteria and “triggering” the growth of beneficial bacteria (152). To be considered a probiotic there has to be scientific evidence (153), nevertheless much of this scientific evidence relies on small sample sized studies that also lack appropriate controls. Thus, there is an urgent need for larger studies and meta-analyses to elucidate the effects of probiotics across different diseases and populations. The most frequently used probiotics, found in fermented dairy such as yoghurt are from *Lactobacillus* and *Bifidobacterium* genera and have shown to modulate both the adaptive immune responses in a strain dependent manner [reviewed extensively in (154)] and dyslipemia in hypercholesterolemic patients (155) but not in normocholesterolemics (156). In experimental mice models, administration of *Akkermansia muciniphila* (an intestinal commensal with anti-inflammatory properties) to *ApoE^−/−^* mice reduced atherosclerosis due to decreased endotoxemia (157) but did not affect neointima formation in *ApoE3-Leiden* mice (158). The effect of probiotics on the adaptive immune response during atherosclerosis remains unexplored.

#### 1.5.2 Prebiotics

Prebiotics are indigestible compounds found in certain foods that selectively nourish beneficial bacteria in the gut improving host wellbeing (159). They can be naturally occurring in fruit, vegetables or whole grains or can be made synthetically. Nondigestible carbohydrates, including oligosaccharides and polysaccharides are the most popular prebiotics (160, 161). They primarily influence the adaptive immune system indirectly promoting probiotics’ growth [extensively summarized in (162)], or via altering metabolite production (p.e. increasing SCFA production). Polyphenols, found in tea, vegetable and cereals modulate the gut microbiota and have been reported to have prebiotic effects via enhancing the growth of probiotic families including *Lactobacilli*, inhibiting pathogenic bacteria such as *E.coli* (163, 164) and increasing SCFA production (164) in mice studies. In fact, tea, also reduced plaque development in *ApoE^−/−^* atherosclerotic mice (165). In humans, several small sized prospective observational studies have associated olive oil, berries, pomegranate juice and cocoa rich in prebiotics with cardioprotective and anti-atherogenic properties (165–171). Nevertheless, in the future larger population studies of the gut microbiota combined with nutritional, genetic and immunephenotyping analysis will be needed to understand thir interaction and how we can utilize it to treat atherosclerosis.

Furthermore, there is an important interaction gut microbiota—anti-atherosclerotic drugs that needs to be studied in more depth to develop more effective and personalized therapies to treat atherosclerosis. For example, statins exhibit prebiotic effects by altering gut microbiota (p.e.: decreasing *Clostridium*) and/or SCFA (171, 172) while at the same time a more diverse gut microbiota leads to a statin greater response (173).

#### 1.5.3 Fecal Microbiota transplantation (FMT)

FMT refers to the transfer of one's own or a donor's faecal sample to a receiver to restore gut microbiome homeostasis. It can be used to improve the gut microbial ecology, however there is an associated risk of transmitting infectious agents and the challenge of donor selection in humans (174, 175). In humans, FMT has been demonstrated to be successful for treating recurrent infections of *Clostridium difficile* (176). Animal experiments using FMT to treat atherosclerosis yielded promising results. FMT from atherogenic mice to WT recipient mice resulted in increased plaque size, increasing circulating innate immune cells and elevated proinflammatory cytokines (177). On the contrary, FMT from healthy mice into an atherosclerotic mouse model significantly decreased disease burden (178). In humans FMT from vegan controls into 20 patients with metabolic syndrome did not affect TMAO or other pro-inflammatory markers (179). While in a more recent clinical trial involving 237 patients that received a FMT from healthy controls demonstrated a significant reduction in cardiovascular risk in patients with metabolic syndrome (180). Additional studies to comprehensively examine the potential efficacy, adverse effects, and translatability of FMT are needed to conclude if this could be a promising therapy to treat atherosclerosis.

#### 1.5.4 Vaccinations

Several studies have demonstrated that B cells in atherosclerotic lesions locally produce antibodies that can react against gut microbe antigens. This may be attributed to bacteria originating from the microbiome present in atherosclerotic plaque (54, 181) or by those present in GALT (132). Notably, these antibodies produced by B lymphocytes in plaques have been reported to exhibit cross-reactivity with epitopes such as oxidised low-density lipoprotein (oxLDL) and cytoskeletal proteins (p.e. transgelin type 1) associated with atherogenesis (182). The development of vaccines that promote atheroprotective antibodies or neutralize atherogenic factors could lower cardiovascular risk.

Binder et al. (183) showed that pneumococcal polysaccharide vaccine (PPV) decreased atherosclerotic lesion formation through a molecular mimicry mechanism between heat-killed *Streptococcus pneumoniae*, found in the microbiome, and oxLDL. This atheroprotective effect was thought to be due to numerous mechanisms including high anti-oxLDL IgM titres blocking uptake of oxLDL by macrophages effectively (184–186). Since then, the link between PPV and cardiovascular events has been controversial. Some trials have provided evidence for an association between PPV and reduced risk of cardiovascular ischaemic events (187, 188) while other studies have not (189–191). However, meta-analyses generally demonstrate significant reductions in CV risk in patients over 65, despite heterogenous samples (192). New clinical studies are ongoing, such as the Australian Study for the Prevention through Immunisation of Cardiovascular Events (AUSPICE), which recruited 4,275 participants, to investigate this further. Although the conclusive findings of this study are pending publication, their preliminary results in smaller subgroups show no changes in CVD and or antibody titers (193, 194).

Additional gut and oral microbial pathogens have been trialed for vaccination. *Porphyromonas gingivalis*, a microbe found in the oral cavity and atherosclerotic plaques has been shown to expedite atherosclerosis via a cross-reactivity mechanism (195, 196). Immunisation against *P. gingivalis* was able to mitigate pathogen-induced atherosclerosis in *ApoE^−/−^* but not in WT mice (197) or on top of statins (198). Moreover, immunization with the outer membrane protein of *Enterobacteriaceae* resulted in decreased inflammatory cells and increased M2 macrophages observed in plaques of both *ApoE^−/−^* and WT mice (181). Human clinical trials are necessary to investigate their translational effect.

In our opinion, vaccines using microbial antigens is a very promising weapon to treat atherosclerosis. However, the challenge of vaccine development includes identifying the correct antigens, predicting efficacy based on findings made on animal models and determining how to measure reduction in plaque size in humans. Successful vaccine development requires the collaboration of multi-disciplinary teams and integration of various techniques.

## 2 Conclusion

The microbiome has emerged as a promising target for addressing atherosclerosis, due to its diverse effects spanning from immunomodulation to metabolite secretion. This review provides a comprehensive overview of how microbiome-targeting mechanisms influence the adaptive immune system, contributing to either atheroprotection or atherogenesis. Whilst the impact of the microbiome on the innate immune system has been studied extensively, the potential role in targeting the adaptive immune system is less understood. Harnessing the microbiome as a therapeutic target can yield multifaceted benefits, including specific and specialized immunomodulation, making it a compelling target for the development of more effective therapies to treat atherosclerosis.
